# Behavioral Efficacy of a Sexual Health Mobile App for Men Who Have Sex With Men: Randomized Controlled Trial of Mobile Messaging for Men

**DOI:** 10.2196/34574

**Published:** 2022-02-02

**Authors:** Patrick Sean Sullivan, Rob Stephenson, Sabina Hirshfield, Cyra Christina Mehta, Ryan Zahn, Jose A Bauermeister, Keith Horvath, Mary Ann Chiasson, Deborah Gelaude, Shelby Mullin, Martin J Downing Jr, Evelyn Jolene Olansky, Sarah Wiatrek, Erin Q Rogers, Eli Rosenberg, Aaron J Siegler, Gordon Mansergh

**Affiliations:** 1 Department of Epidemiology Rollins School of Public Health Emory University Atlanta, GA United States; 2 Department of Systems, Population, and Leadership School of Nursing University of Michigan Ann Arbor, MI United States; 3 Special Treatment and Research Program Department of Medicine The State University of New York Downstate Health Sciences University Brooklyn, NY United States; 4 Division of Infectious Diseases Department of Medicine Emory University School of Medicine Atlanta, GA United States; 5 Department of Biostatistics and Bioinformatics Rollins School of Public Health Emory University Atlanta, NY United States; 6 Department of Family and Community Health School of Nursing University of Pennslyvania Philadelphia, PA United States; 7 Department of Psychology San Diego State University San Diego, CA United States; 8 Department of Epidemiology Mailman School of Public Health Columbia University New York, NY United States; 9 Division of Infectious Diseases Department of Medicine Columbia University Irving Medical Center New York, NY United States; 10 HIV Research Branch Division of HIV Prevention Centers for Disease Control and Prevention Atlanta, GA United States; 11 Department of Psychology Lehman College City University of New York Bronx, NY United States; 12 Social & Scientific Systems, Inc DLH Holdings Company Atlanta, GA United States; 13 Department of Epidemiology School of Public Health University at Albany, State University of New York Albany, NY United States

**Keywords:** HIV prevention, mHealth, tool, video, randomized clinical trial, app, prevention, HIV, PrEP, STI, testing, behavior, efficacy, men who have sex with men, MSM, sexuality, gay, bisexual, United States

## Abstract

**Background:**

Gay, bisexual, and other men who have sex with men (GBMSM) face the highest burden of HIV in the United States, and there is a paucity of efficacious mobile health (mHealth) HIV prevention and care interventions tailored specifically for GBMSM. We tested a mobile app combining prevention messages and access to core prevention services for GBMSM.

**Objective:**

This study aims to measure the efficacy of the Mobile Messaging for Men (M-cubed) app and related services to increase HIV prevention and care behaviors in diverse US GBMSM.

**Methods:**

We conducted a randomized open-label study with a waitlist control group among GBMSM in 3 groups (low-risk HIV-negative group, high-risk HIV-negative group, and living-with-HIV [LWH] group) recruited online and in venues in Atlanta, Detroit, and New York City. Participants were randomly assigned to receive access to the app immediately or at 9 months after randomization. The app provided prevention messages in 6 domains of sexual health and offered ordering of at-home HIV and sexually transmitted infection test kits, receiving preexposure prophylaxis (PrEP) evaluations and navigation, and service locators. Serostatus- and risk-specific prevention outcomes were evaluated at baseline, at the end of the intervention period, and at 3, 6, and 9 months after the intervention period.

**Results:**

In total, 1226 GBMSM were enrolled and randomized; of these 611 (49.84%) were assigned to the intervention group and 608 (99.51%) were analyzed, while 615 (50.16%) were assigned to the control group and 612 (99.51%) were analyzed. For high-risk GBMSM, allocation to the intervention arm was associated with higher odds of HIV testing during the intervention period (adjusted odds ratio [aOR] 2.02, 95% CI 1.11-3.66) and with higher odds of using PrEP in the 3 months after the intervention period (aOR 2.41, 95% CI 1.00-5.76, *P*<.05). No changes in HIV prevention or care were associated with allocation to the intervention arm for the low-risk HIV-negative and LWH groups.

**Conclusions:**

Access to the M-cubed app was associated with increased HIV testing and PrEP use among high-risk HIV-negative GBMSM in 3 US cities. The app could be made available through funded HIV prevention providers; additional efforts are needed to understand optimal strategies to implement the app outside of the research setting.

**Trial Registration:**

ClinicalTrials.gov NCT03666247; https://clinicaltrials.gov/ct2/show/NCT03666247

**International Registered Report Identifier (IRRID):**

RR2-10.2196/16439

## Introduction

The ambitious goal of the US Ending the HIV Epidemic Initiative to reduce new HIV diagnoses by 90% by 2030 [1] will not be reached without reducing new HIV infections in gay, bisexual, and other men who have sex with men (GBMSM). GBMSM comprised 54% of annual new HIV diagnoses in 2019 in the United States [2] and face the highest burden of HIV in the United States [3]. However, there are few efficacious or promising mobile health (mHealth) HIV prevention and care interventions tailored for subgroups of GBMSM [4,5]. New HIV prevention tools should address the needs of GBMSM, young GBMSM aged 15-24 years (YGBMSM) [6], and YGBMSM of color, for whom HIV incidence is the highest [7-9]. Currently, HIV prevention services are underused by GBMSM: in 2018, just over half (56%) reported being tested in the past 12 months, high proportions (76% of HIV-positive and 66% of HIV-negative GBMSM) reported recent condomless receptive anal intercourse, and few (<20%) reported preexposure prophylaxis (PrEP) use [10,11]. Statistical models demonstrate that high levels of coverage of prevention services will be required to substantially reduce HIV incidence among GBMSM [12,13] and increased use of routine prevention services (eg, HIV testing) might increase uptake of biomedical interventions, such as PrEP [14]. A package of HIV prevention interventions is needed to maximally reduce the risks of infection, and uptake of multiple prevention services by a substantial proportion of the GBMSM population is needed to reduce HIV incidence among GBMSM.

A growing body of research has suggested that mobile phone apps are a promising environment to offer tailored and on-demand prevention services for GBMSM [5,6,15-19]. GBMSM are open to receiving prevention information and resources via mobile apps [20]. Communicating prevention messages through mobile apps (mobile messaging) might enhance intervention uptake, because it allows messaging to a wide audience of GBMSM, including rural GBMSM, who use sexual health services and PrEP at lower rates than GBMSM in urban areas [10,11,21]. Younger GBMSM might be especially interested in using mobile technology to receive health information [22].

HIV prevention and care interventions for GBMSM have most often been provided through in-person sessions with behavior change interventions, are often focused on specific subgroups of GBMSM (eg, high-risk HIV-negative episodic substance-using GBMSM, Black GBMSM with HIV-negative or unknown HIV serostatus, HIV-positive clinic patients) [23] and often target only 1 element of comprehensive prevention—for example, condom use, medication adherence, or PrEP uptake [4]. In a serostatus-neutral framework [24], we must evaluate interventions that address multiple prevention behaviors (eg, HIV testing, sexually transmitted infection [STI] testing, condom use, PrEP uptake, and medication adherence), many of which are relevant to GBMSM with HIV and those at risk for HIV infection.

To address these needs, we adapted an existing HIV prevention app designed for GBMSM, HealthMindr, to add and evaluate tailored prevention messaging [25-27]. The methods for the app content development specific to this study have been described elsewhere [28]. We aimed to develop a mobile app that can address multiple prevention and care needs for GBMSM, both those with HIV and those at risk for HIV acquisition (ie, HIV negative). We developed messaging for HIV-negative GBMSM that would be relevant for both high- and low-risk MSM. Messages were drawn from existing mHealth interventions [29,30] and adapted through a theory-driven approach with stakeholder input, as previously described [28]. Messages were provided through in-app content and videos. We used the core HealthMindr app [25,26] to offer a suite of prevention services, including self-screening for HIV and STI risk; a scheduling and reminder system for routine HIV and STI testing; a PrEP eligibility screener; a nonoccupational postexposure prophylaxis (nPEP) risk assessment tool; an ordering platform for delivery of at-home HIV- and STI-screening kits and of condoms and lubricants; and service locators for HIV and STI testing, PrEP, nPEP, and HIV treatment and care. The app included branding associating the app with Emory University. Content was frozen during the trial. Participants accessed the app from Apple App Store or Google Play Store and received a study-specific activation code.

We aimed to evaluate the use and efficacy of the combined prevention messaging and app service components as a public health intervention strategy to reduce risks of HIV acquisition and improve HIV care outcomes among GBMSM. We hypothesized that exposure to the message delivery platform within a comprehensive HIV prevention app would result in improvements in participants’ self-reported sexual health, prevention, and care behaviors, compared to GBMSM without access to the app.

## Methods

### Study Design

Data were collected from January 24, 2018, to October 31, 2019. The methods for the study have been previously described [28] and are summarized here. Eligibility criteria are summarized in Table 1. Men were classified as (1) HIV seropositive, (2) HIV seronegative at high risk (any anal sex not protected by condoms or not taking PrEP, as prescribed, in the past 3 months), and (3) HIV seronegative at low risk (anal sex protected by condoms in the past 3 months or taking PrEP, as prescribed, in the past 3 months, ie, adherent PrEP users). Owning a cell phone was also required, and smartphone literacy was thus a de facto eligibility criterion. This research was reviewed and approved by the Emory University institutional review board (protocol IRB00087684) on June 13, 2016. We collected individual informed consent in person at the study enrollment visit. Consenting participants were randomly assigned to either the immediate intervention group or the waitlist control group in a 1:1 ratio within 36 strata based on the 3 serostatus/risk groups, 3 cities, race/ethnicity (non-Hispanic White vs other race/ethnicity), and phone type (Android vs iOS) [31]. Participants in the intervention group received one 2-sentence message every other day and a 1-minute video weekly, in addition to access to HIV and STI kit ordering and informational resources.

At in-person baseline visits, participants assigned to the intervention group downloaded and received orientation to the study app and received access to the mobile app and prevention messages based on their risk profiles. Participant contact and retention activities were supported through the Emory Study Management and Retention Tool (SMART). The intervention was provided over 3 months, after which the app was deactivated. Waitlist control participants were not provided access to the intervention app at the baseline visit but continued to receive quarterly surveys. An overview of the study timeline and complete survey instruments have been published and are available online [28].

There were multiple prevention outcomes, which varied among the 3 risk groups, as previously reported [28]. The specific outcomes and measures used are summarized in Table 2.

**Table 1:** Eligibility criteria for a randomized controlled trial of the Mobile Messaging for Men intervention in Atlanta, Detroit, and New York City (2018-2019).

Criterion	Eligible level
Age (years)	≥18
Sex at birth	Assigned as male
Current gender identity	Male
Sexual risk behaviors	Reports anal intercourse with a male partner in the past 12 months
City of residence	Resident of Atlanta, Detroit, or New York City
Other eligibility criteria	Plans to stay in the city area for the next 9 months Owns and uses an Android or iOS smartphone Is able to read and understand English without assistance

**Table 2:** Outcomes and measures used in the evaluation of Mobile Messaging for Men, a mobile health intervention to promote HIV prevention and care behaviors among GBMSM^a^ in Atlanta, Detroit, and New York City (2018-2019).

Behavior	Group assessed	Definition
STI^b^ testing	All GBMSM	STI test^c^ in the 3 months before the survey
HIV testing	Low-risk HIV-negative GBMSM High-risk HIV-negative GBMSM	Self-reported^d^ HIV test in the 3 months before the survey
Current PrEP^e^ use	Low-risk HIV-negative GBMSM High-risk HIV-negative GBMSM	Self-reported^d^ being on PrEP as of the day of the survey
PrEP adherence	All low- and high-risk HIV-negative GBMSM reporting PrEP use	Self-reported^d^ taking at least 25/30 daily pills in the prior 30 days
Current ART^f^ use	All LWH^g^ GBMSM	Self-reported^d^ being on ART as of the day of the survey
ART adherence	All LWH GBMSM on ART	Self-reported^d^ taking ART, as prescribed, on ≥25 of the past 30 days
Anal sex not protected by PrEP or condoms	Low-risk HIV-negative GBMSM High-risk HIV-negative GBMSM	Self-reported anal sex with a main or casual partner when the participant was not on PrEP AND the insertive partner did not use a condom from start to finish^d^
Anal sex not protected by PrEP or condoms	LWH GBMSM	Self-reported anal sex with a main or casual partner when the insertive partner did not use a condom from start to finish^d^

^a^GBMSM: gay, bisexual, and other men who have sex with men.

^b^STI: sexually transmitted infection.

^c^Includes Mobile Messaging for Men care kit orders with results.

^d^Self-report measures were previously validated as part of the American Men’s Internet Survey [32].

^e^PrEP: preexposure prophylaxis.

^f^ART: antiretroviral therapy.

^g^LWH: living with HIV.

### Data Analysis

Analyses were stratified by the baseline HIV status/risk group: high-risk HIV-negative, low-risk HIV-negative, and living with HIV (LWH) groups. Counts and relative frequencies were used to describe sociodemographics and sexual history at baseline by intervention status (intervention group vs control group) and HIV status/risk group, and chi-square tests assessed for differences in these characteristics by intervention status (Table 3). To account for within-person repeated measures and randomization strata, separate mixed-effect logistic models assessed for association over time of the intervention with each outcome.

A post hoc analysis was conducted to compare control participants with intervention participants who had at least 30 minutes of intervention use in order to assess the potentially mediating effect of the time spent on the app; 30 minutes was the estimated time required to read and rate all written messages and view the video messages. A second post hoc analysis was conducted because not all participants who ordered and received at-home STI test kits returned them. In this second analysis, we defined the intention to test for STIs as either ordering an STI test kit through the mobile app or reporting having taken an STI test. Result reporting was prepared in accordance with Consolidated Standards of Reporting Trials (CONSORT) guidelines [33].

**Table 3:** Sociodemographic and baseline behavioral characteristics of MSM^a^ in the Mobile Messaging for Men study sample (2018-2019).

Characteristic		Overall (N=1220)	High-risk^b^ HIV-negative (n=427)			Low-risk^c^ HIV-negative (n=410)			HIV-positive (n=383)	
		n (%)	n (%)	*P* value^d^ (intervention vs control)	n (%)		*P* value (intervention vs control)	n (%)		*P* value (intervention vs control)
**Race/ethnicity^e^**										
	MSM of color	709 (58.11)	205 (48.0)	.89	204 (49.8)		.92	300 (78.3)		.84
	White	510 (41.80)	222 (52.0)	—^f^	206 (50.2)		—	82 (21.4)		—
**Age (years)**										
	18-29	448 (36.72)	210 (49.2)	.13	180 (43.9)		.89	58 (15.1)		.58
	≥30	772 (63.28)	217 (50.8)	—	230 (56.1)		—	325 (84.9)		—
**Education level^g^**										
	≤High school diploma/General Education Development (GED) test	176 (14.42)	54 (12.6)	.96	31 (7.6)		.46	91 (23.8)		.76
	Some post–high school education	387 (31.72)	128 (29.9)	—	98 (23.9)		—	161 (42.0)		—
	4-year college degree	377 (30.90)	141 (33.0)	—	158 (38.5)		—	78 (20.4)		—
	Some graduate education	277 (22.70)	102 (23.9)	—	123 (30.0)		—	52 (13.6)		—
**City/metropolitan statistical area**										
	Atlanta	473 (38.77)	140 (32.8)	.98	145 (35.4)		.99	188 (49.1)		.98
	Detroit	333 (27.29)	155 (36.3)	—	121 (29.5)		—	57 (14.9)		—
	New York City	414 (33.93)	132 (30.9)	—	144 (35.1)		—	138 (36.0)		—
**Baseline outcome variables**										
	STI^h^ testing (3 months)	—	165 (38.4)	.18	254 (61.9)		.27	271 (71)		.09
	HIV testing (3 months)	—	162 (37.9)	.40	259 (63.2)		.74	—		—
	Current PrEP^i^ use	—	22 (5.1)	.08	222 (54.1)		.21	—		—
	PrEP adherence (≥25/30 days)^j^	—	11 of 22 (50.0)	.36	212 of 222 (95.5)		.99	—		—
	Current ART^k^ use	—	—	—	—		—	368 (96.1)		.99
	ART adherence (≥25/30 days)^j^	—	—	—	—		—	335 of 368 (91.0)		.06
	Unprotected anal sex (3 months)^l^	—	392 (91.8)	.37	51 (12.4)		.73	98 (25.6)		.01
	Engagement in care (3 months)^m^	—	45 (10.5)	.10	196 (47.8)		.42	251 (65.5)		.06

^a^MSM: men who have sex with men.

^b^High risk (condomless/PrEP-less anal sex, past 3 months).

^c^Low risk (no condomless/PrEP-less anal sex, past 3 months).

^d^*P* value for chi-square testing (difference between intervention and control arms).

^e^One HIV-positive participant did not report race/ethnicity.

^f^Not applicable.

^g^Two high-risk HIV-negative and one HIV-positive participants did not report education level.

^h^STI: sexually transmitted infection.

^i^PrEP: preexposure prophylaxis.

^j^Among current users.

^k^ART: antiretroviral therapy.

^l^Condomless and PrEP-less anal sex for HIV-negative and condomless/detectable viral load for HIV-positive users.

^m^Engagement in PrEP or ART care.

## Results

### Recruitment and Randomization

**Figure 1:**
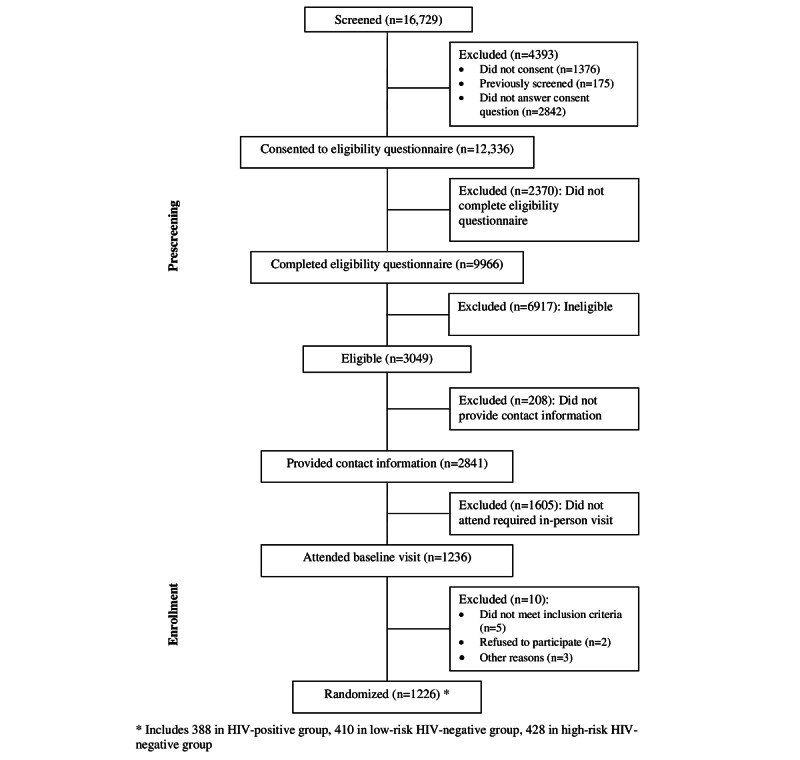
CONSORT diagram for the M-cubed randomized controlled trial among GBMSM (screening through randomization). CONSORT: Consolidated Standards of Reporting Trials; GBMSM: gay, bisexual, and other men who have sex with men; M-cubed: Mobile Messaging for Men.

From January 24, 2018, to November 2, 2018, a total of 9966 GBMSM were assessed for eligibility for the randomized controlled trial; reasons for lack of eligibility are shown in Figure 1. Specifically, 2841 (28.51%) of 9966 GBMSM screened were eligible and provided contact information, of which 1236 (43.51%) attended a baseline study visit, 1226 (99.19%) consented and were randomized to intervention (n=611, 49.84%) and control (n=615, 50.16%) arms (Figure 1).

### Sample Characteristics

**Figure 2:**
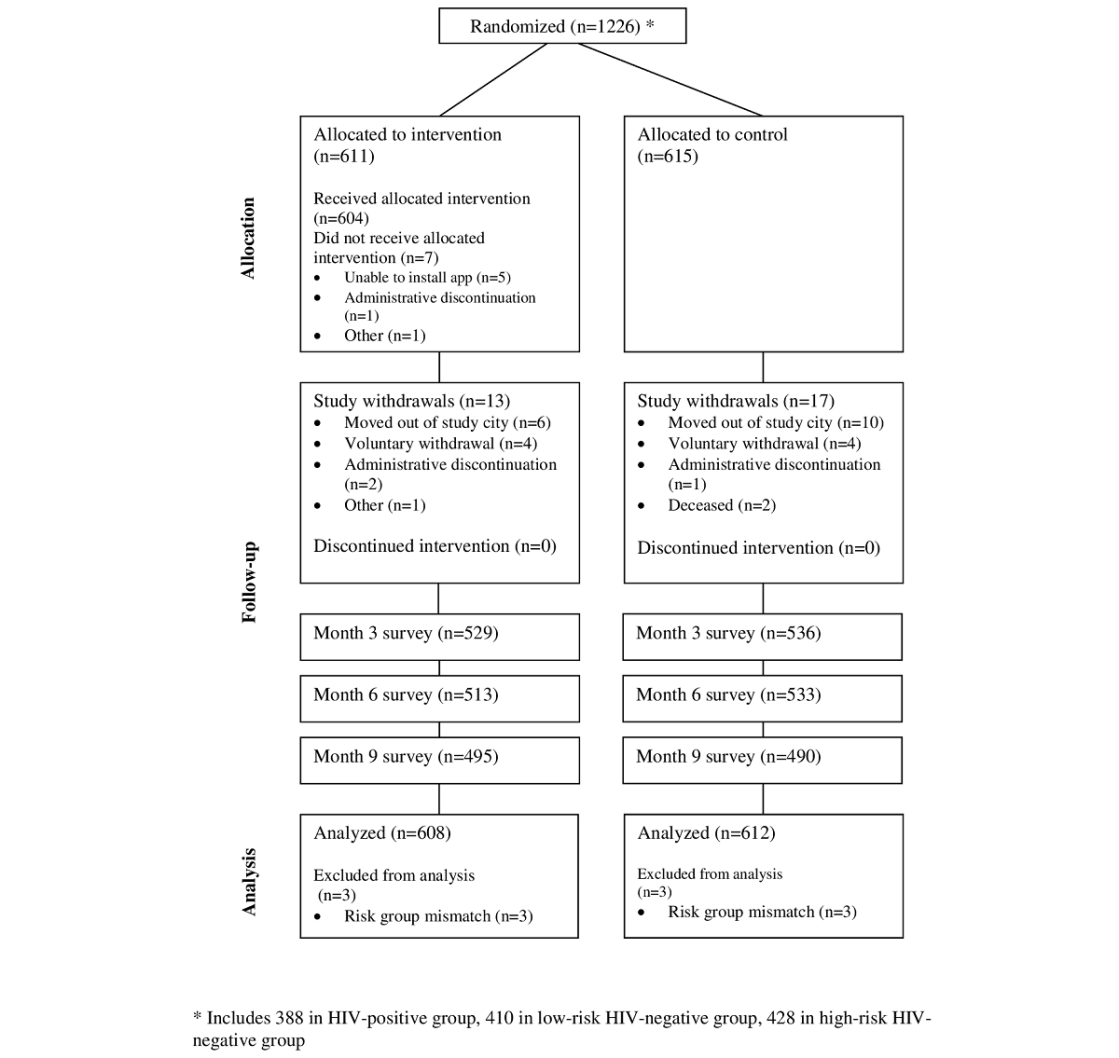
CONSORT diagram for a randomized controlled trial of the M-cubed intervention for GBMSM (randomization through analysis). CONSORT: Consolidated Standards of Reporting Trials; GBMSM: gay, bisexual, and other men who have sex with men; M-cubed: Mobile Messaging for Men.

The analytic sample comprised 1220 GBMSM enrolled in the study, including 608 (49.84%) randomized to the intervention arm and 612 (50.16%) randomized to the control arm (Figure 2). There was no failure of randomization within the 3 risk groups, except for unprotected anal intercourse among LWH GBMSM (Table 2). Most participants were GBMSM of color (n=378 [30.98%] Black/African American, n=183 [15%] Hispanic/Latino, n=148 [12.13%] other/mixed), were aged ≥30 years (772/1220 [63.28%]), and had at least a 4-year college education (654/1220 [53.61%]). Other indicators of risk and prevention services did not differ between control and intervention arms (data not shown). Self-reported levels of use of prevention and care services were often less than recommended by the Centers for Disease Control and Prevention (CDC). High-risk HIV-negative GBMSM reported moderate levels of STI (165/427, 38.6%) and HIV (162/427, 37.9%) testing in the 3 months before baseline; only 22 of 427 (5.1%) reported using PrEP, and adherence to PrEP was low (11/22, 50%, reported ≥25/30 pills in the prior 30 days). A high proportion (392/427, 91.8%) reported sex not protected by PrEP or condoms in the past 3 months. Among low-risk HIV-negative GBMSM, baseline use of STI (254/410, 61.9%) and HIV (259/410, 63.2%) testing was higher; in addition, 222 of 410 (54.1%) reported being on PrEP, with high (212/222, 95.5%) adherence. Many HIV-negative GBMSM were designated as low risk because they were on PrEP; routine PrEP care includes HIV and STI testing. Among LWH GBMSM, most (271/383, 70.8%) reported recent STI testing, being on antiretroviral therapy (ART; 368/383, 96.1%) and high ART adherence (335/368, 91%). About 1 in 4 reported anal sex not protected by condoms in the past 3 months (98/383, 25.6%), and most (251/383, 65.5%) reported engaging in HIV care during the prior quarter.

Retention was 1065 of 1226 (86.87%) at 3 months, 1046 of 1226 (85.32%) at 6 months, and 985 of 1226 (80.34%) at 9 months. Factors associated with retention were consistent across time points: retention was higher among participants who were White non-Hispanic, had more education, were employed full-time, and were not homeless. At the 9-month follow-up, higher retention was also associated with gay/homosexual identity (vs bisexual or other identities) and online recruitment (vs venue-based recruitment).

### Intervention Efficacy

**Figure 3:**
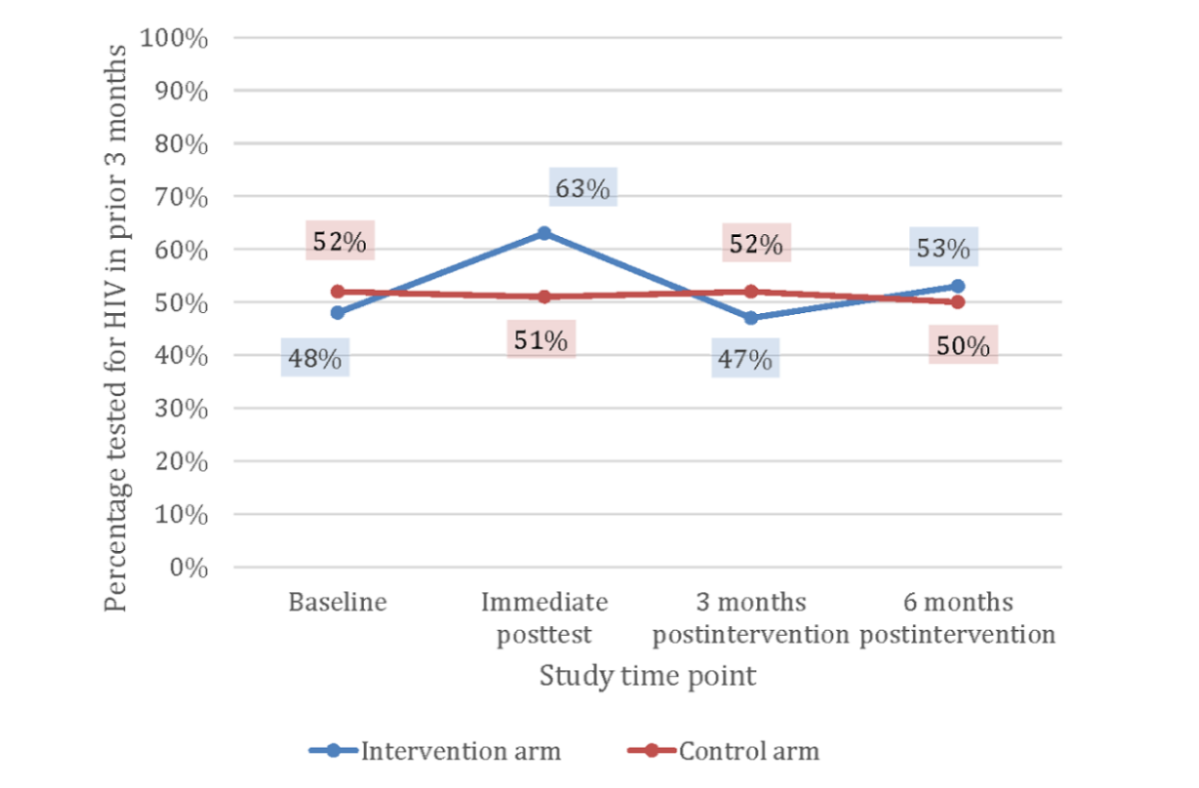
HIV testing of high-risk GBMSM, reported by study time point and randomized allocation, in Atlanta, Detroit, and New York City (2018-2019). GBMSM: gay, bisexual, and other men who have sex with men.

**Figure 4:**
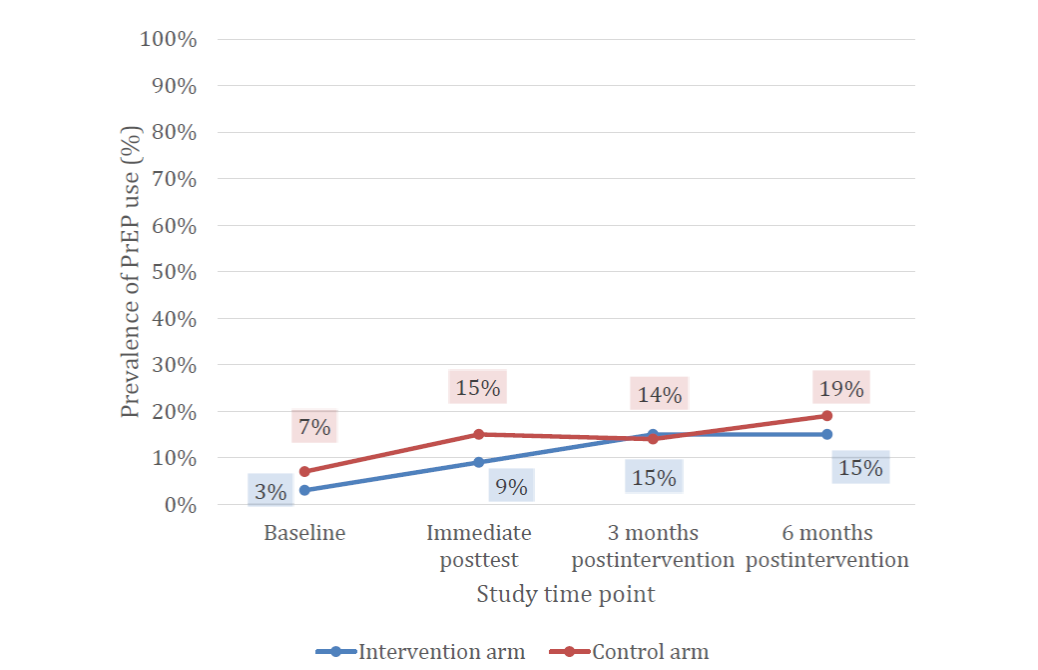
PrEP use among GBMSM who reported anal intercourse not protected by condoms or PrEP at baseline, reported by study time point and randomized allocation, in Atlanta, Detroit, and New York City (2018-2019). GBMSM: gay, bisexual, and other men who have sex with men; PrEP: preexposure prophylaxis.

Intervention efficacy was analyzed by HIV status/risk group (Table 4). Among high-risk HIV-negative GBMSM, the odds of HIV testing (Figure 3) were higher at the immediate postintervention time point and the odds of current PrEP use (Figure 4) were higher at the 3-month postintervention time point, but not at 6 or 9 months postintervention (Table 4). Of the high-risk GBMSM in the intervention arm who tested for HIV in the immediate postintervention period (n=126), 16 (12.7%) used an at-home HIV self-test kit provided through the app. As shown in Figure 4, although the initial prevalence of PrEP use was nonsignificantly lower in the intervention group at baseline, PrEP use rose from 3% at baseline to 15% by the 3-month follow-up assessment and remained at 15% through the 6-month follow-up assessment. Although odds ratio (OR) estimates comparing intervention to control for PrEP adherence were >1.0 at all postintervention time points for high-risk GBMSM, they were not statistically significant, given the small number of high-risk HIV-negative GBMSM taking PrEP.

For low-risk HIV-negative GBMSM, there were no changes in study outcomes associated with assignment to the intervention. For LWH GBMSM, STI testing was significantly lower in the immediate posttest period in the intervention group compared to the control group.

**Table 4:** Modeled behavioral measures at baseline, immediate posttest, and 3- and 6-month postintervention follow-up assessments for intervention efficacy of the Mobile Messaging for Men mobile app among MSM^a^ by HIV status/risk group (N=1220).

Behavioral measure		High-risk HIV-negative MSM (n=427)				Low-risk HIV-negative MSM (n=410)				HIV-positive MSM (n=383)		
		Intervention group, M%^b^ (95% CI)	Control group, M% (95% CI)	aOR^c^ (95% CI)	Intervention group, M% (95% CI)		Control group, M% (95% CI)	aOR (95% CI)	Intervention group, M% (95% CI)		Control group, M% (95% CI)	aOR (95% CI)
**STI^d^ testing (3 months)**												
	Baseline	35 (28-43)	41 (34-49)	—^e^	58 (50-66)		64 (55-71)	—	76 (68-82)		68 (59-75)	—
	Immediate posttest	42 (35-50)	47 (39-55)	1.07 (0.61-1.87)	61 (53-69)		56 (48-65)	1.53 (0.87-2.70)	65 (56-73)		72 (63-79)	*0.49* *(0.26-0.94)^f^*
	3 months postintervention	39 (31-47)	45 (37-53)	1.01 (0.58-1.76)	63 (54-71)		56 (48-64)	1.65 (0.97-2.81)	72 (63-79)		59 (50-68)	1.19 (0.63-2.24)
	6 months postintervention	37 (30-45)	42 (34-50)	1.06 (0.64-1.74)	57 (48-66)		58 (49-66)	1.22 (0.77-1.94)	71 (62-78)		63 (54-72)	0.96 (0.53-1.73)
**HIV testing (3 months)**												
	Baseline	48 (39-57)	52 (44-60)	—	73 (65-80)		71 (63-78)	—	—		—	—
	Immediate posttest	63 (55-71)	51 (42-59)	*2.02* *(1.11-3.66)^f^*	75 (67-81)		65 (57-73)	1.41 (0.75-2.64)	—		—	—
	3 months postintervention	47 (39-55)	52 (44-60)	0.98 (0.55-1.75)	69 (61-77)		66 (58-73)	1.03 (0.57-1.86)	—		—	—
	6 months postintervention	53 (45-61)	50 (42-58)	1.34 (0.80-2.25)	68 (60-76)		68 (59-75)	0.90 (0.54-1.52)	—		—	—
**Current PrEP^g^ use**												
	Baseline	3 (2-7)	7 (4-11)	—	55 (45-64)		49 (39-58)	—	—		—	—
	Immediate posttest	9 (6-14)	15 (11-21)	1.26 (0.48-3.32)	52 (42-62)		49 (39-58)	0.89 (0.60-1.31)	—		—	—
	3 months postintervention	15 (10-21)	14 (10-20)	*2.41* *(1.00-5.76)^f^*	52 (42-61)		48 (39-59)	0.88 (0.63-1.23)	—		—	—
	6 months postintervention	15 (11-21)	19 (14-26)	1.67 (0.81-3.47)	51 (42-61)		49 (39-58)	0.85 (0.66-1.10)	—		—	—
**PrEP adherence (≥25/30 days)**												
	Baseline	35 (10-71)	62 (37-82)	—	95 (89-98)		95 (88-98)	—	—		—	—
	Immediate posttest	78 (54-92)	80 (61-91)	2.72 (0.26-28.15)	91 (82-95)		84 (74-91)	1.66 (0.39-7.02)	—		—	—
	3 months postintervention	84 (64-94)	74 (55-87)	5.47 (0.58-51.75)	92 (84-96)		85 (75-91)	1.84 (0.43-7.80)	—		—	—
	6 months postintervention	85 (64-94)	92 (77-98)	1.38 (0.13-14.28)	91 (82-95)		92 (83-96)	0.88 (0.21-3.66)	—		—	—
**Current ART^h^ use**												
	Baseline	—	—	—	—		—	—	98 (94-99)		98 (95-99)	—
	Immediate posttest	—	—	—	—		—	—	97 (92-77)		98 (94-99)	0.83 (0.17-4.15)
	3 months postintervention	—	—	—	—		—	—	98 (94-99)		96 (92-98)	2.43 (0.56-10.55)
	6 months postintervention	—	—	—	—		—	—	96 (91-98)		96 (91-98)	1.11 (0.38-3.30)
**ART adherence (≥25/30 days)**												
	Baseline	—	—	—	—		—	—	96 (92-98)		91 (85-95)	—
	Immediate posttest	—	—	—	—		—	—	93 (87-96)		91 (85-95)	0.58 (0.18-1.85)
	3 months postintervention	—	—	—	—		—	—	93 (87-96)		92 (85-95)	0.40 (0.13-1.18)
	6 months postintervention	—	—	—	—		—	—	92 (86-96)		92 (86-96)	0.43 (0.16-1.14)
**Unprotected anal sex/all partners (3 months)^i^**												
	Baseline	94 (90-97)	92 (87-95)	—	14 (9-20)		15 (10-21)	—	21 (16-28)		34 (27-42)	—
	Immediate posttest	78 (71-84)	73 (65-80)	0.93 (0.39-2.24)	23 (16-31)		28 (21-37)	0.79 (0.38-1.64)	20 (14-27)		20 (14-28)	1.93 (0.92-4.06)
	3 months postintervention	72 (64-79)	76 (67-82)	0.58 (0.25-1.35)	22 (15-30)		29 (22-38)	0.75 (0.38-1.49)	15 (10-22)		19 (14-27)	1.46 (0.68-3.15)
	6 months postintervention	70 (61-77)	66 (58-74)	0.82 (0.40-1.67)	27 (20-35)		26 (19-35)	1.11 (0.62-2.01)	18 (12-25)		18 (12-25)	1.98 (0.96-4.07)
**Engagement in PrEP/ART care (3 months)**												
	Baseline	8 (5-13)	13 (9-19)	—	49 (40-59)		46 (37-55)	—	70 (63-77)		61 (53-68)	—
	Immediate posttest	16 (11-22)	18 (13-25)	1.38 (0.62-3.04)	46 (37-56)		45 (36-55)	0.91 (0.54-1.55)	61 (53-69)		58 (50-66)	0.75 (0.40-1.39)
	3 months postintervention	17 (12-24)	15 (10-22)	1.90 (0.89-4.04)	47 (37-57)		44 (35-54)	0.97 (0.60-1.57)	68 (60-76)		56 (48-64)	1.11 (0.60-2.04)
	6 months postintervention	17 (12-24)	20 (14-27)	1.37 (0.73-2.56)	42 (33-52)		43 (34-53)	0.82 (0.55-1.22)	64 (55-72)		55 (47-63)	0.95 (0.54-1.65)

^a^MSM: men who have sex with men.

^b^M%, model-based mean probability of reporting behavior.

^c^aOR: adjusted odds ratio (for intervention vs control change from baseline; regression models include independent variables of the intervention group [intervention, control], survey time point [baseline, immediate posttest, 3- and 6-month postintervention follow-up], and their interaction).

^d^STI: sexually transmitted infection.

^e^Not applicable.

^f^*P*<.05.

^g^PrEP: preexposure prophylaxis.

^h^ART: antiretroviral therapy.

^i^Condomless/PrEP-less anal sex for HIV-negative MSM and condomless/detectable viral load for HIV-positive MSM.

### Posthoc Analyses of Intervention Effects by Key Demographic Variables and Time Spent on App and STI Testing

Given the potential importance of race/ethnicity, age group, and education level as potential moderators of intervention effects, we conducted stratified analyses of the outcome variables for each of the 3 HIV status/risk groups for the following subgroups: non-Hispanic White versus others; age 18-29 versus ≥30 years; and <4-year college degree versus 4-year degree or more. No meaningful difference in outcomes was identified among stratum-specific estimates. Further, a sensitivity analysis assessed the time spent on the mobile app over the 3-month intervention (control vs ≥30 minutes of intervention use) for the 3 HIV status/risk groups. No meaningful or significant difference in outcomes was identified.

In a posthoc analysis, the intention to test for STIs (ie, ordering an STI test kit or reporting having had an STI test) was higher in all HIV-negative participants (low-risk HIV-negative: aOR 3.1, 95% CI 1.7-5.6; high-risk HIV-negative: aOR 3.5, 95% CI 2.0-6.2; all HIV-negative regardless of risk group: aOR 3.4, 95% CI 2.3-5.1).

## Discussion

### Principal Results

We tested an app-based intervention to provide messages to promote HIV prevention and care and provide access to core HIV and STI prevention services to GBMSM. The results showed significant increases in HIV screening during the period of app use among high-risk HIV-negative GBMSM. The results also indicated increased prevalence of PrEP use among high-risk HIV-negative GBMSM in the 3-month period after app use. We identified no protective changes in study outcomes for low-risk HIV-negative GBMSM or LWH GBMSM. Given the high baseline PrEP use and adherence among low-risk HIV-negative GBMSM who did not report recent anal sex not protected by condoms and high levels of ART use and the high baseline adherence among LWH GBMSM, there was little room for improvement statistically among these groups on key outcome variables. We recognize, however, that the vulnerability of GBMSM may change over time (ie, seasons of risk [34]), so the utility of the app and its messaging might change over time for individual men.

We developed our intervention in light of 3 principles. First, we worked from the paradigm of integrated HIV prevention and care continua. Although the HIV status was determined at the beginning of the trial, when the app is used in practice, HIV testing will be the starting point for both continua. Thus, the intervention could serve GBMSM who do not know their HIV status [35]. Second, we sought to develop an intervention that could be offered to all GBMSM rather than to demographic or risk subsets of men. Third, we sought to develop an intervention that could be scaled to provide intervention content to large numbers of men without the need for a large staff of interventionists. A free, downloadable app that disseminates one 2-sentence message every other day and a 1-minute video weekly might be easily incorporated into a user’s life without requiring the time and travel often required of more traditional, in-person interventions. Further, mobile app interventions are a potentially low-cost strategy from the perspective of health departments and community-based organizations (CBOs) that may choose to implement them in their jurisdictions.

We view our mixed efficacy outcomes from several perspectives. The fact that exposure to the app was associated with a doubling of HIV-testing behaviors and of prevalence of PrEP use among high-risk HIV-negative GBMSM suggests that the messages and app services could be a scalable, effective resource for GBMSM who engage in anal intercourse that is not protected by condoms or PrEP. Large national surveys of GBMSM suggest that 47%-53% of HIV-negative GBMSM would fall into this category [36,37]; considering the number of high-risk HIV-negative GBMSM in the United States [38], the potential user base for these services is about 1,800,000-2,000,000 US GBMSM.

The effect sizes for uptake of HIV screening and PrEP use were both about 2. However, the baseline levels of HIV screening and PrEP use were quite different. For high-risk GBMSM, the CDC recommends HIV testing at least annually, and in the intervention arm, nearly two-thirds reported HIV testing in the immediate posttest assessment period (tested in the prior 3 months). However, despite the same relative increase in PrEP use, only 1 in 6 (17%) high-risk GBMSM was taking PrEP at the 3-month postintervention time point. This suggests that additional and more intensive intervention services will likely be required for high-risk PrEP-eligible men who do not initiate PrEP within 6 months of starting the use of the Mobile Messaging for Men (M-cubed) app.

We also recognize that increases in PrEP usage and HIV testing associated with allocation to the app were short lived. The timing of the effects makes sense: the support for HIV testing provided during the period of app use included ordering at-home kits and navigating to testing locations; the significant increase in testing was observed during the period when men had access to the app, and about 1 in 7 high-risk men who tested for HIV used a mailed-out HIV self-test kit provided through the study. For PrEP, after the decision to start PrEP, there is a long process of making an appointment, undergoing lab tests, filling a prescription, and starting the medication; this outcome was higher in the 3 months after the period of app use. In practice, exposure to the app would not be limited to 3 months, and it is a separate question as to whether changes in behavior would be sustained over time with ongoing exposure to the app. Such a question could be answered in an implementation study.

Our approach was developed with an eye toward being able to reach large numbers of GBMSM with a basic package of prevention services. Given that there was efficacy for some outcomes, there are important questions about how the intervention might be made available to GBMSM who would be likely to benefit from it [39]. Most HIV prevention services are currently provided and funded through CBOs and health departments. Although some CBOs and health departments might have the technical capacity to support app-based prevention services, this capacity is likely quite limited. A currently underway randomized study of an efficacious eHealth intervention (Keep It Up [40]) will examine the outcomes of centralized versus decentralized implementation approaches, and the results of the study may inform how the M-cubed intervention could be most effectively implemented [41].

### Limitations

Our study was subject to several limitations. Although we used recruitment quotas to guide recruitment and achieved a balanced sample with respect to the 3 HIV status/risk groups, race/ethnicity, and recruitment venues, our sample was skewed to men with higher education. These selection biases threaten the external generalizability of the study; it is possible that the intervention might have had salutary effects for low-risk HIV-negative GBMSM or LWH GBMSM with lower education. However, although not powered for secondary comparisons, our post hoc stratified analyses did not suggest differential effects by educational attainment. Second, PrEP use in our sample was high at baseline (about 29% among all HIV-negative GBMSM), which is unique in large samples of GBMSM to date. For example, current PrEP use was reported by less than 20% of respondents to the American Men’s Internet Survey (a large, national sample of US GBMSM) in 2017 [10] and by 25% of GBMSM who participated in 23 cities in the 2017 National HIV Behavioral Surveillance study [37]. Over half of GBMSM in our study who were not on PrEP reported HIV testing in the 3 months before enrollment; this is similar to the proportion who tested for HIV in the 12 months before interviews in the 2016 AMIS survey [11]. Third, outcomes were self-reported and so might be subject to misclassification because of social desirability bias [42]. Fourth, our sample was restricted to GBMSM recruited online; this might introduce selection bias, in that men recruited through physical venues, such as sex venues, might have higher or lower levels of risk. Lastly, our sample was limited to GBMSM in 3 urban areas of the United States who reported sex with a man in the past year; patterns of risk behaviors and use of prevention services are known to be different in other urban areas or among rural GBMSM [11,21]. The level of ART use in our LWH MSM was high, offering limited opportunity for improvement. Future studies of the app could condition enrollment on the need for support for ART initiation or maintenance for LWH MSM.

The next steps for the study include secondary analysis of extensive app usage data or *paradata* [43] in terms of understanding the ratings of each message and the time spent using various optional aspects of the app descriptively [44]. We will also conduct further assessments of the potential mediation of outcome effects by app usage to help direct future prevention research and program implementation of mobile apps. Based on our data, the app offered benefits to GBMSM who reported recent anal sex not protected by condoms or PrEP but not to HIV-negative men who did not report sex not protected by condoms of PrEP or LWH men. Therefore, we will consider how the app can be updated and implemented [39,45] for GBMSM engaging in sex not protected by condoms or PrEP and for LWH MSM. Because we found that the app is a successful way to increase the intention to test for STIs and to distribute at-home STI self-test kits, the kits were often not returned and additional research is needed to understand the barriers to returning STI specimen collection kits. If this barrier can be overcome, the M-cubed app might also effectively support increased STI testing for some groups of GBMSM.

### Conclusion

We sought to produce and evaluate a mobile app with prevention messaging and services to support comprehensive HIV prevention and care with low levels of prevention staff interaction for all GBMSM, regardless of HIV status or current risk behaviors. Such a serostatus-neutral approach, if implemented successfully, would offer advantages in terms of cost efficiency and minimization of segmentation of GBMSM by serostatus. Future work with the M-cubed app should focus on implementing the app for GBMSM who are likely to benefit from it based on their current behaviors and evaluating methods of implementation of the app through public or publicly supported prevention providers [39].

## Appendix

Multimedia Appendix 1 CONSORT-eHEALTH checklist (V 1.6.1).

## Abbreviations

aOR: adjusted odds ratio
ART: antiretroviral therapy
CBO: community-based organization
CDC: Centers for Disease Control and Prevention
CONSORT: Consolidated Standards of Reporting Trials
GBMSM: gay, bisexual, and other men who have sex with men
LWH: living with HIV
M-cubed: Mobile Messaging for Men
mHealth: mobile health
MSM: men who have sex with men
nPEP: nonoccupational postexposure prophylaxis
OR: odds ratio
PrEP: preexposure prophylaxis
SMART: Study Management and Retention Tool
STI: sexually transmitted infection
YGBMSM: young gay, bisexual, and other men who have sex with men
